# Localization-specific distributions of protein pI in human proteome are governed by local pH and membrane charge

**DOI:** 10.1186/s12860-019-0221-4

**Published:** 2019-08-20

**Authors:** Atsushi Kurotani, Alexander A. Tokmakov, Ken-Ichi Sato, Vasily E. Stefanov, Yutaka Yamada, Tetsuya Sakurai

**Affiliations:** 1 RIKEN Center for Sustainable Resource Science, 1-7-22, Suehiocho, Yokohama, Kanagawa 230-0045 Japan; 2 Faculty of Life Sciences, Kyoto Sangyo University, Kamigamo-motoyama, Kita-ku, Kyoto, 603-8555 Japan; 3 Department of Biochemistry, Saint-Petersburg State University, Universitetskaya 7/9, Saint-Petersburg, 199034 Russia; 4 Interdisciplinary Science Unit, Multidisciplinary ScienceCluster, Research and Education Faculty, Kochi University, 200 Otsu, Monobe, Nankoku, Kochi 783-8502 Japan

**Keywords:** Bioinformatics, Human proteome, Protein pI, Protein localization, Regression analysis

## Abstract

**Background:**

Whole-proteome distributions of protein isoelectric point (pI) values in different organisms are bi- or trimodal with some variations. It was suggested that the observed multimodality of the proteome-wide pI distributions is associated with subcellular localization-specific differences in the local pI distributions. However, the factors responsible for variation of the intracellular localization-specific pI profiles have not been investigated in detail.

**Results:**

In this work, we explored proteome-wide pI distributions of 32,138 human proteins predicted to reside in 10 subcellular compartments, as well as the pI distributions of experimentally observed lysosomal and Golgi proteins. The distributions were found to differ significantly, although all of them adhered to the major recurrent bimodal pattern. Grossly, acid-biased and alkaline-biased patterns with various minor statistical features were observed at different subcellular locations. Bioinformatics analysis revealed the existence of strong statistically significant correlations between protein pI and subcellular localization. Most markedly, protein pI was found to correlate positively with nuclear and mitochondrial locations and negatively with cytoskeletal, cytoplasmic, lysosomal and peroxisomal environment. Further analysis demonstrated that subcellular compartment-specific pI distributions are greatly influenced by local pH and organelle membrane charge. Multiple nonlinear regression analysis identified a polynomial function of the two variables that best fitted the mean pI values of the localization-specific pI distributions. A high coefficient of determination calculated for this regression (*R*^2^ = 0.98) suggests that local pH and organelle membrane charge are the major factors responsible for variation of the intracellular localization-specific pI profiles.

**Conclusions:**

Our study demonstrates that strong correlations exist between protein pI and subcellular localization. The specific pI distributions at different subcellular locations are defined by local environment. Predominantly, it is the local pH and membrane charge that shape the organelle-specific protein pI patterns. These findings expand our understanding of spatial organization of the human proteome.

**Electronic supplementary material:**

The online version of this article (10.1186/s12860-019-0221-4) contains supplementary material, which is available to authorized users.

## Background

Physicochemical properties of proteomes and sub-proteomes vary significantly, reflecting differences in environmental conditions and evolutionary trends of organism taxonomy. Several reports addressed proteome-wide distributions of protein isoelectric point (pI) that can be reliably calculated from raw amino acid sequence in a good agreement with experimentally observed values [1, 2]. Availability of whole-genome sequences allowed comparative proteome-wide studies of protein pI distributions. Initially, it was found that the proteome-wide pI distributions are bimodal, with acidic and alkaline peaks, in several bacterial strains [3–5]. This general bimodality was supposed to result from discrete acidic and basic pKas of different amino acid side chains. It was suggested that the low representation of proteins with neutral pIs reduces protein aggregation at physiological intracellular pH, as the proteins are least soluble at their pI values. Indeed, protein solubility was shown to correlate directly with the content of charged residues in the human proteins produced in a cell-free bacterial system, and the lowest rate of soluble expression was observed for the proteins with pI 7.0–7.5 expressed in this system [6, 7]. It was further found that cytosolic and integral membrane proteins have pI distributions corresponding to the two observed modes; cytoplasmic proteins exhibited a distinct clustering at pI 5.0 to 6.0, and integral membrane proteins clustered at around pI 8.5 to 9.0 [8]. Furthermore, global analysis of complete predicted proteomes using the “theoretical 2D gels” revealed that the proteins of membrane proteomes are generally more basic than those represented in non-membrane proteomes [9]. Subsequent whole-proteome studies demonstrated that the pI distributions of eukaryotic proteins are generally trimodal. It was suggested that the third peak can be related to the emergence of nuclear proteins in eukaryotes. Indeed, nuclear proteins were found to have a broad distribution encompassing the range from pI 4.5 to 10.0 that may account for the third mode found in eukaryotes [8]. Some additional peaks, for instance, a minor peak at around pI = 11.5, were also observed in the proteome-wide pI distributions [10, 11], further suggesting the existence of distinct subcellular localization-specific protein pI profiles.

Differences in the localization-specific pI distributions were linked to the facts that milieu pH values differ in various subcellular compartments, and that the proteins with pI values different from the pH of their milieu are more soluble and have an increased folding stability. Indeed, there is a tendency for protein pI values averaged over a subcellular location to differ from the local pH. On the other hand, it was reported that the averaged values of local pI distributions match experimentally determined intra-organellar pH estimates across different subcellular compartments of the yeast cell and further hypothesized that protein pI might have co-evolved with subcellular organelle pH to optimize protein function [12]. However, it is not clear whether the observed correlation between protein pI and organelle pH is conserved across species. More recent analysis of multiple proteomes in various biological species ranging from bacteria to eukaryotes could not reveal a statistically significant correlation between the subcellular pI distributions and pH of the compartments where these proteomes were located [13]. In addition, several works indicated that the pI multimodality phenomenon is not related to subcellular localization or taxonomy and may result just from discrete pKa values for different amino acids [10, 14, 15]. In this connection, the evidence has been presented for the adaptation of protein pH dependence, but not protein pI, to subcellular pH. It was found that the average pH of maximal stability, rather than the average pI of proteins in a subcellular compartment, correlates with subcellular pI [15–17]. Importantly, previous studies demonstrated that the pI value and pH optimum for protein stability and activity can be quite different [18, 19].

In the present work, we revisited relationships between protein pI and subcellular localization. The distributions of calculated pI values were investigated in the human proteome across 10 distinct cellular compartments, including cytoplasmic, nuclear, membrane, mitochondrial, lysosomal, cytoskeletal, reticular, peroxisomal, Golgi and extracellular localizations. Also, pI distributions of the experimentally observed human proteins in the lysosomal and Golgi compartments were examined. To disclose the factors related to variation of the intracellular localization-specific pI patterns, correlations of protein pI with local pH and organelle membrane charge were analyzed and multiple regression analysis was carried out. The results of our study demonstrate that strong correlations, defined by organelle pH and membrane charge, exist between protein pI and subcellular localization.

## Results

### Overview of the human proteome-wide pI distribution

**Fig. 1:**
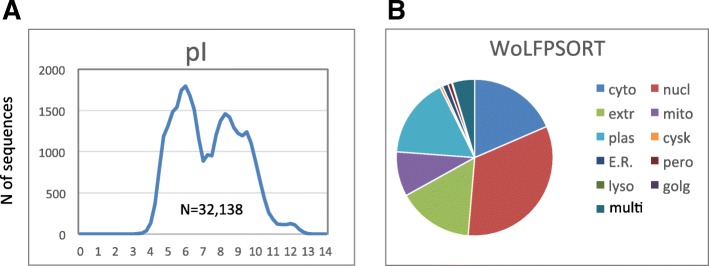
Distributions of protein pIs and subcellular localization in the human proteome. **a** Distribution of calculated pI values of 32,138 predicted human proteins. **b** Distribution of protein subcellular localization, as predicted by the WoLF PSORT algorithm. The panel shows relative contents of proteins in 10 major subcellular locations and the proteins predicted to locate in multiple compartments (denoted as “multi”)

The distribution of pI values determined for 32,138 predicted proteins in the human proteome is presented in Fig. 1a. It is essentially bimodal with the major acidic and alkaline peaks at pI 6.0 and 8.25, respectively. Markedly, the peaks are not Gaussian, and the distribution, as a whole, displays a number of minor statistical features, such as peak shoulders, sub-peaks and minor peaks. These features are further scrutinized in the “Distribution profiling” section. The proteins of the analyzed dataset were predicted to reside, by the WoLF PSORT algorithm, in the multiple subcellular compartments, such as cytoplasmic, nuclear, membrane, mitochondrial, lysosomal, cytoskeletal, reticular, peroxisomal, Golgi and extracellular locations (Fig. 1b). The protein pI distributions in each of these compartments are presented in the following section. The most abundant localizations included nuclear, cytoplasmic, plasma membrane, extracellular and mitochondrial compartments; they comprised more than 90% of all dataset proteins. Around 4% of the proteins were predicted to reside in multiple compartments (denoted as “multi” in Fig. 1b).

### Localization-specific pI distributions

**Fig. 2:**
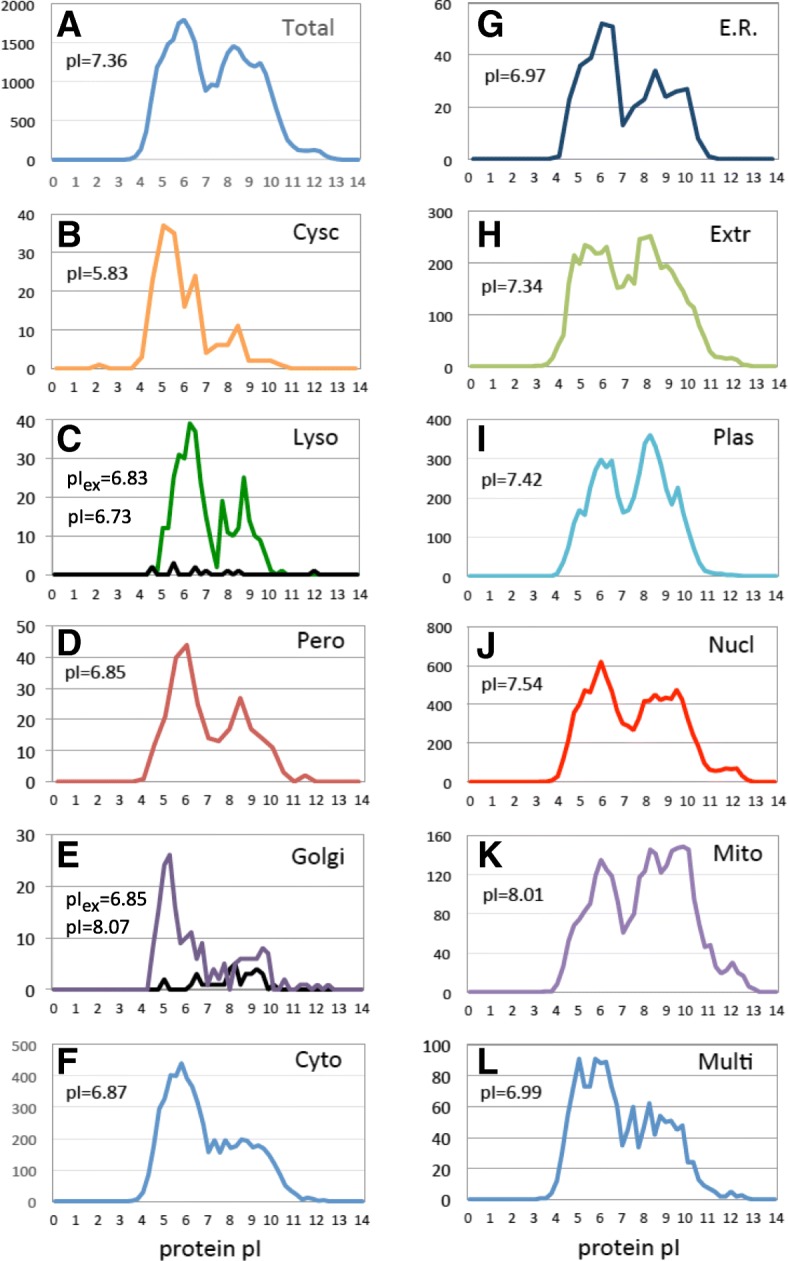
pI distributions of proteins in different subcellular compartments. Panel **a** shows, for comparison, the whole-proteome pI distribution. Panels **b**-**k** present the pI distributions at the indicated locations, and panel **l** shows the distribution of proteins predicted to locate in multiple cellular compartments. The mean pI values of the distributions are indicated in the panels. pI_ex_ and pI in panels **c** and **e** refer to the mean pI values calculated for datasets of experimental and predicted lysosomal and Golgi proteins

Next, the pI distribution profiles were built for the proteins predicted to reside in different subcellular compartments. All of the local distributions followed the major bimodal pattern observed in the whole proteome pI profile, as presented in Fig. 2. However, the relative content of acidic and alkaline proteins varied greatly in the distributions. In addition, each of the local pI distributions displayed various minor statistical features. The distributions are largely overlapping, as it is evident from the graphs presented in Fig. 2, reflecting the fact that all the subcellular compartments contain proteins with various pI values ranging from about pI 4.0 to 12.0. The calculated mean pI values for the proteins localized in different subcellular compartments differed from the averaged value determined for the whole proteome (pI = 7.36), and they varied from pI 5.83, for the cytoskeletal proteins, to 8.01, for the proteins predicted to localize in the mitochondrial compartment. Of note, the distribution profile of the proteins predicted to reside in multiple compartments (Fig. 2l) resembled the pattern of the proteome-wide pI distribution, as it could be expected considering compositional diversity of this protein subset.

### pI distributions in the subsets of experimentally determined lysosomal and Golgi proteins

The predicted lysosomal and Golgi subsets were quite small, demanding alternative verification of the results obtained for these intracellular locations. For this purpose, we analyzed pI distributions in the subsets of proteins experimentally detected in the lysosomal and Golgi fractions (Additional files 1 and 2: Tables S1 and S2). The pI distribution of the experimentally observed lysosomal proteins was found to be substantially acidic, with the mean pI value of 6,83 (Fig. 2c). The value was close to that found for the subset of bioinformatically predicted lysosomal proteins, confirming acidic bias of the lysosomal pI distribution. On the other hand, the pI distributions of the experimentally observed and bioinformatically predicted Golgi proteins differed significantly, with the mean pI values of 6,85 and 8.07, respectively (Fig. 2e). The pH distribution of the experimentally observed Golgi proteins was used in the following analysis because a previous study has also reported an acidic bias of Golgi proteins [13]. However, the exact value of the mean pI was not provided in that work.

### pI distribution profiling and proteome-wide correlations

**Fig. 3:**
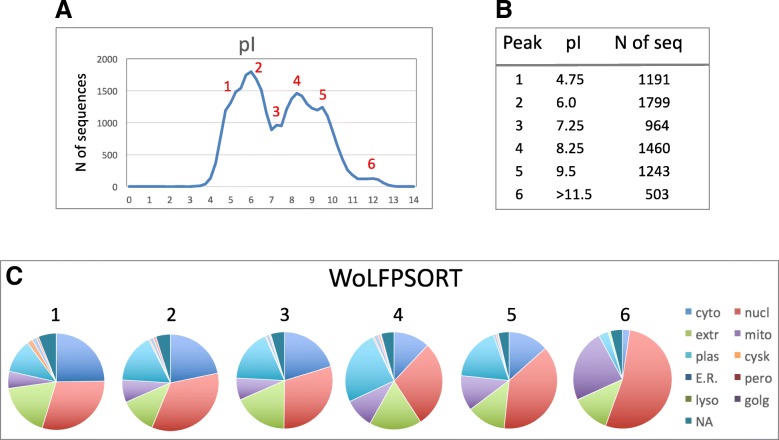
Profiling of subcellular localization and function across the whole-proteome pI distribution. Panels **a** and **b** define the six reference points where protein localization was analyzed. Panel **c** presents the contents of proteins with different localization at each reference point

**Fig. 4:**
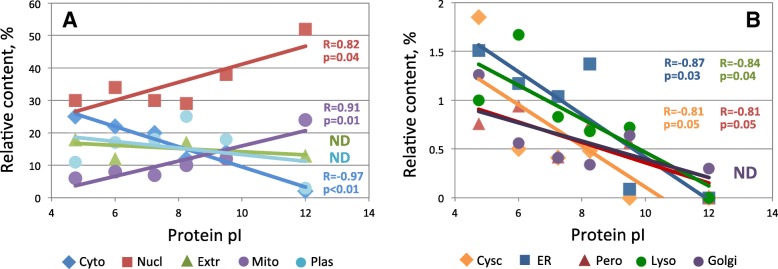
Proteome-wide correlations between protein pI and subcellular localization. Panels **a** and **b** present the correlations for the high and low abundant localizations, respectively. Pearson’s pairwise correlation coefficients and their statistical significance are indicated in the panels

The bimodal whole-proteome pI profile (Fig. 1a) comprised various minor traits that were thought to stem from the different localization-distribution patterns presented in Fig. 2. To reinforce this assumption, protein subcellular localization patterns were analyzed at several reference points of the whole-proteome pI distribution. The six pI values, coinciding with shoulders, peaks and sub-peaks in the whole-proteome pI distribution, were designated as the reference points, as indicated in Fig. 3a, b. Subcellular localization patterns differed greatly in the reference points (Fig. 3c). To further highlight these differences and reveal major tendencies, we built the cumulative linearized plots showing the changes in protein localization along the whole-proteome pI distribution. A strong positive correlation was observed between protein pI and propensity for nuclear and mitochondrial localization, whereas a negative correlation was evident for cytoplasmic, cytoskeletal, endoplasmic reticulum, peroxisomal and lysosomal proteins (Fig. 4). All of the observed relationships were statistically significant at the level of *p* < 0.05, as determined by calculating two-tailed probability values (see “Methods”).

### The factors behind pI distribution differences

**Fig. 5:**
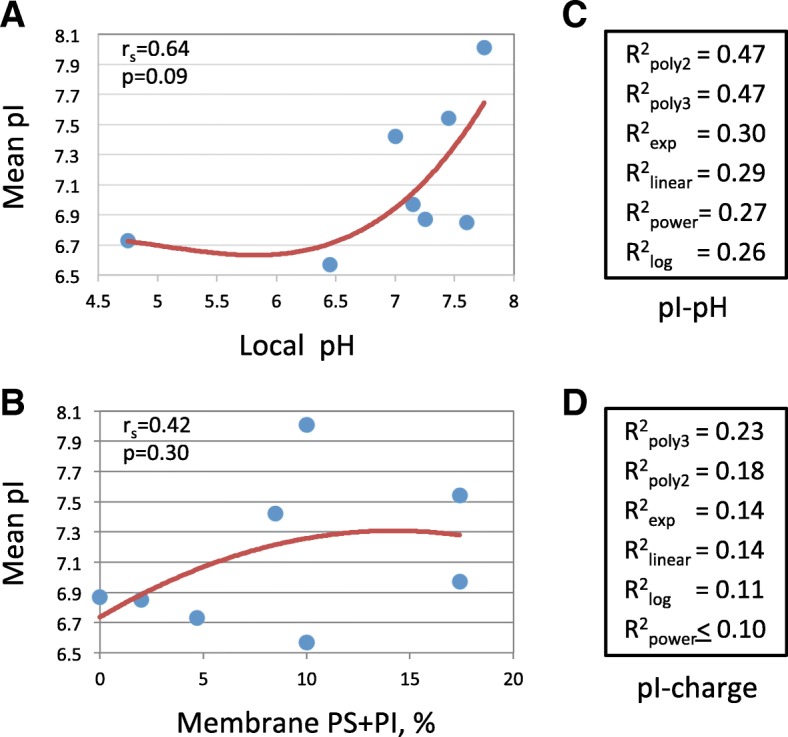
Regression analysis of relationships between protein pI, intra-organelle pH and membrane charge. Spearman’s correlation coefficients and their *p*-values are indicated in panels **a** and **b**. Coefficients of determination for the polynomial (degree 2 and 3), exponential, linear, power, and logarithmic regressions are presented in panels **c** and **d**

The protein pI distributions varied significantly at different subcellular locations (Fig. 2). We suggested that specific environments of subcellular compartments could be responsible for the observed differences in the pI distributions. On this premise, the relationships between the mean distribution pI and the intra-compartment pH, as well as the compartment membrane charge, as designated in Table 1, were scrutinized. Of note, the subset of cytoskeletal proteins was found to be extremely acid-biased in a sharp difference to the subset of cytoplasmic proteins located in the same subcellular compartment (Fig. 2b). The reason for this difference is not clear, however, one can speculate that due to polymerization, cytoskeletal proteins cannot be considered as truly soluble. Thus, cytoskeletal proteins were excluded from this analysis. The correlations of mean pI with compartment pH and membrane charge were essentially nonlinear (Fig. 4a, b), so the nonparametric Spearman’s correlation coefficients were determined for these relationships. This test revealed the lack of statistically significant, at the level of *p* < 0.05, correlations between mean local pI and compartment pH, as well as membrane charge. Further regression analysis of the relationships between the mean pI and any of the two variables failed to identify an approximation function that had a statistically significant coefficient of determination. Altogether, more than 100 various functions were tried for the best fitting of analyzed data. The major regressions included linear, polynomial, power, logarithmic and exponential functions (Fig. 5c, d).

**Table 1:** Intra-organelle pH and membrane charge

Compartment	pH (2009)	pH (2015)	Charge, %
Cytoplasm	7.3	7.2	0.0
Nucleus	7.7	7.2	17.4
E.R.	7.1	7.2	17.4
MX	7.5	8.0	10.0
Golgi	6.6	6.0–6.7	10.0
Peroxisome	8.2	7.0	2.0
Lysosome	4.8	4.7	4.7
Membrane	–	–	8.5

### Multiple regression analysis

**Fig. 6:**
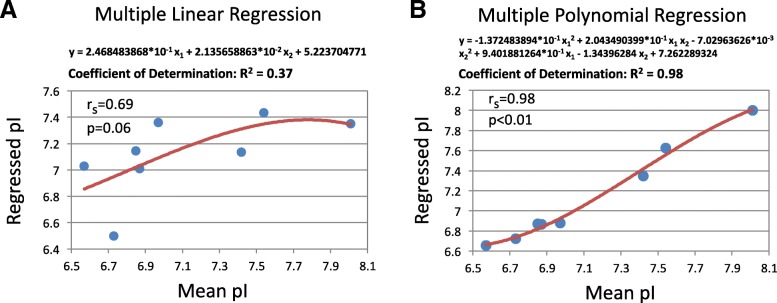
Multiple regression analysis of relationships between protein pI, intra-organelle pH and membrane charge. The variables X_1_ and X_2_ in the linear (**a**) and polynomial (**b**) regressions, indicated above the graphs, refer to organelle pH and membrane charge, correspondingly. Spearman’s coefficients and their p-values were determined for correlations between the mean pIs of the localization-specific distributions, as presented in Fig. 2, and the pI values calculated with the use of the indicated regression functions

Next, we performed multiple linear and non-linear regression analyses in search of a composite function of the two variables, compartment pH and membrane charge, that approximates localization-specific mean pI with a statistically significant determination coefficient. Although the best linear fitting for multiple regression had a better coefficient of determination than any of the individual linear regressions (*R*^2^ = 0.37 vs 0.29 and 0.14), it was not statistically significant, as it could be judged from the Spearman’s correlation coefficient (r_s_ = 0.69; *p* = 0.06) (Fig. 6a). Remarkably, multiple nonlinear regression identified a polynomial approximation function that fitted the analyzed data set with a very high coefficient of determination (*R*^2^ = 0,98). The Spearman’s correlation coefficient between the mean distribution pI and the regressed pI values was statistically significant at the level of *p* < 0.01 (Fig. 6b). Altogether, these results indicate that local pH and membrane charge can account, when combined, for major variance of the mean pI distribution values observed at different subcellular locations.

## Discussion

In the present work, we analyzed localization-specific pI distribution patterns of proteins in the human proteome. The latest update of human genome data was used in this analysis. Previously, it was reported that eukaryotic whole-proteome pI distributions are generally trimodal, reflecting differences in the cytoplasmic, nuclear and plasma membrane sub-proteomes [8]. However, our present study shows that the human proteome pI distribution is essentially bimodal with various minor statistical features (Figs. 1a, 3a). Although some of these features, for instance, a minor peak at pI> 11.0, were mentioned in previous studies, they were not associated with specific subcellular localizations.

**Fig. 7:**
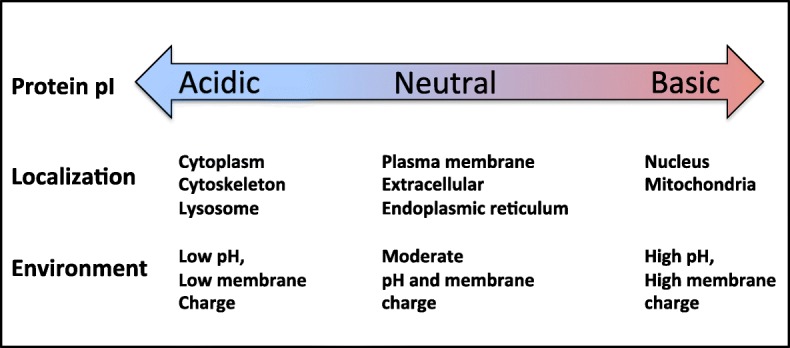
Major relationships between protein pI and subcellular localization revealed in this study

In this study, calculative and predictive bioinformatics algorithms were used to assign the pI values and subcellular localizations to all proteins in the human proteome. The WoLF PSORT tool was employed to predict subcellular localization of proteins. Based on these assignments, localization-specific pI profiles were built and further analyzed. This analysis revealed a number of statistically significant correlations between protein pI and subcellular localization. Specifically, a strong positive correlation was observed between protein pI and propensity for nuclear and mitochondrial localization, and a negative correlation for cytoplasmic, cytoskeletal, endoplasmic reticulum, peroxisomal and lysosomal proteins (Fig. 4). The proteome-wide relationships between protein pI and subcellular localization are summarized in Fig. 7. These findings are largely consistent with the results of a previous bioinformatics study of multiple proteomes. It was demonstrated that the proteomes of the cytoplasm, lysosomes and cytoskeleton are acidic, whereas those of the plasma membrane and mitochondria are basic [13].

The results obtained by the subcellular localization profiling (Figs. 3 and 4) help to explain how the different localization-specific distribution patterns form the whole-proteome pI profile. For example, the existence of an acidic shoulder in the major acidic peak (pI = 4.75, reference point 1) can be attributed to over-representation of low pI cytoplasmic and extracellular proteins, whereas the most alkaline distinct sub-peak (pI> 11.5, reference point 6, Additional file 3: Table S3) of the whole-genome distribution is mainly composed of nuclear and mitochondrial proteins (Figs. 3 and 4). Previously, this peak was observed in some organisms [10, 11], however its protein composition was not investigated in detail. We further scrutinized this peak and found that most of the nuclear proteins in the extra-alkaline subset bear the nucleolar localization signal, NoLS, and are functionally involved in RNA processing, ribosomal biogenesis, chromatin dynamics and transcription (data not shown). Of note, the majority of proteins in the extra-alkaline subset still lack functional annotation, demanding their further characterization.

The main result of this study is the finding that organelle-specific protein pI patterns are defined largely by local pH and membrane charge. First, our analysis revealed the lack of statistically significant correlation between mean pI and intra-organelle pH (Fig. 5a, c). This result agrees well with several previous reports [10, 13, 15]. Next, we noticed that the pI distribution of plasma membrane proteins was alkaline-biased (Fig. 2i), and suggested that plasma membrane environment might be related to this fact. We further hypothesized that the membrane charge might be a factor related to the observed variation of intracellular localization-specific pI patterns and examined the correlation between mean distribution pIs and membrane charges. To our knowledge, this kind of analysis has not been performed before. No statistically significant correlation was revealed by this analysis between membrane charge and mean pI (Fig. 5b, d). Finally, multiple regression analysis, which is used to disclose the relation between several variables, identified a polynomial approximation function that best fitted the analyzed data set with a very high coefficient of determination (Fig. 6b), indicating that local pH and membrane charge together are the major factors defining intracellular localization-specific pI values.

## Conclusions

Our work provides the most comprehensive analysis yet of subcellular localization- specific pI distributions in the human proteome. The major findings of this study are concisely presented in Fig. 7. In sum, protein pI correlates positively with nuclear and mitochondrial localizations and negatively with cytoplasmic, cytoskeletal, peroxisomal, lysosomal and endoplsmic localizations. The key factors that influence subcellular localization-specific pI distributions are local pH and membrane charge. These findings contribute to our understanding of spatial organization of the human proteome.

## Methods

### Data sets

The complete human proteome dataset was constructed using the proteome resource available at ftp://ftp.ncbi.nlm.nih.gov/genomes/Homo_sapiens/protein/. The redundancy check was carried out using the CD-HIT tool [20] to remove amino acid sequences with more than 90% identity. The sequences containing less than 50 amino acids were also filtered out. The total number of entries in the final whole-proteome dataset was 32,138.

A subset of extra-alkaline human proteins was extracted from the complete human proteome dataset. It contained amino acid sequences with the calculated pI value > 11.5. The total number of sequences in the extra-alkaline subset was 503 (Additional file 3: Table S3).

A dataset of experimentally observed lysosomal proteins was constructed using the Human Lysosome Gene Database [21] available at http://lysosome.unipg.it/index.php#results. Filtering of redundant sequences has not been performed. Only the proteins with the established intra-lysosomal localization were included in the dataset. The total number of amino acid sequences in the dataset of experimental lysosomal proteins was 355 (Additional file 1: Table S1).

A dataset of experimentally observed Golgi proteins was constructed using the Human Protein Atlas [22, 23] available from https://www.proteinatlas.org/. The total number of amino acid sequences in the dataset of experimentally observed Golgi proteins was 196 (Additional file 2: Table S2).

### Calculation and prediction of protein properties

Protein pI values were calculated using the free ProtParam tool [2] provided at the ExPASy server (https://web.expasy.org/protparam/).

Protein localization was predicted with the WoLF PSORT [24], Advanced Protein Subcellular Localization Prediction Tool, freely downloadable from the GenScript server (https://www.genscript.com/wolf-psort.html).

Nucleolar localization signals (NoLS) in the amino acid sequences of extra-alkaline subset were identified with the NoD, Nucleolar Localization Sequence Detector, predictive tool available online (http://www.compbio.dundee.ac.uk/www-nod/) [25, 26].

### Intra-organelle pH and membrane charge

The intra-organelle pH values were extracted from the two previous publications [17, 27]. Although largely consistent with each other, they differed at some subcellular locations (Table 1). The extracted values were averaged and the average values were used in the following correlation analysis.

The membrane charges (Table 1) were assigned to different subcellular compartments on the basis of previously reported relative contents of charged phospholipids, such as phosphatidylserine (PS) and phosphatidylinositol (PI) [28]. In addition, the content of charged phospholipids in peroxisomal membranes was extracted from [29].

### Correlation analysis and statistics

The calculated protein pI values were correlated with several predicted or previously reported parameters, such as subcellular localization, intra-organelle pH, and organelle membrane charge, using the pairwise regression analysis. The strength and direction of the observed correlations was evaluated by calculating Pearson’s (linear) or Spearman’s (nonlinear) correlation coefficients. The statistical significance of the correlation coefficients was determined by calculating two-tailed probability values (p), given the correlation coefficient value (r) and sample size (n), with the level of statistical significance *p* < 0.05. Calculations of Pearson’s correlation coefficients and *p*-values were carried out using the statistics calculators available online at https://www.danielsoper.com/statcalc/default.aspx. Spearman’s correlation coefficients and their p-values were calculated using the statistics tool available online at https://www.socscistatistics.com/tests/spearman/default2.aspx.

### Regression analysis

Linear and nonlinear regression analyses were carried out to find the best approximation functions that characterize relation between two or several variables with the highest coefficient of determination. For the relation between two variables (Fig. 5), only coefficients of determination for the major regressions, such as linear, polynomial, power, logarithmic, and exponential, were presented. For the multiple regression analysis, which investigates relation between several variables, both the regression functions and coefficients of determination were indicated (Fig. 6). The regression function was selected according to the least squares’ fitting. More than 100 various functions were tested for the best fitting of analyzed datasets. Regression analysis was performed using an online statistics tool available at http://www.xuru.org/Index.asp.

## Additional files

Additional file 1: **Table S1.** A dataset of experimentally observed lysosomal proteins. (XLSX 52 kb)

Additional file 2: **Table S2.** A dataset of experimentally observed Golgi proteins. (XLSX 43 kb)

Additional file 3: **Table S3.** A dataset of extra-alkaline proteins in the human proteome. (XLSX 59 kb)

## Data Availability

A human proteome dataset analyzed in this study was constructed using the proteome resource available from ftp://ftp.ncbi.nlm.nih.gov/genomes/Homo_sapiens/protein/. A dataset of experimentally observed lysosomal proteins was extracted from the Human Lysosome Gene Database available at http://lysosome.unipg.it/index.php#results, and a dataset of experimentally observed Golgi proteins was composed using the Human Protein Atlas at https://www.proteinatlas.org/. All data generated during this study are included in this published article and its supplementary information files.

## Abbreviations

ER: Endoplasmic reticulum
NoLS: Nucleolar Localization Signal
pI: isoelectric point
PI: Phosphatidylinositol
pKa: acid dissociation constant
PS: Phosphatidylserine
WoLF PSORT: Advanced Protein Subcellular Localization Prediction Tool
